# Comparison of Two Commercially Available Interferon-γ Release Assays for T-Cell-Mediated Immunity and Evaluation of Humoral Immunity against SARS-CoV-2 in Healthcare Workers

**DOI:** 10.3390/diagnostics13040637

**Published:** 2023-02-08

**Authors:** Alexandra Lochmanová, Jan Martinek, Hana Tomášková, Hana Zelená, Kersten Dieckmann, Evelin Grage-Griebenow, Eduard Ježo, Jaroslav Janošek

**Affiliations:** 1 Public Health Institute Ostrava, 70200 Ostrava, Czech Republic; 2 Institute of Laboratory Medicine, Faculty of Medicine, University of Ostrava, 70300 Ostrava, Czech Republic; 3 Department of Epidemiology and Public Health, Faculty of Medicine, University of Ostrava, 70300 Ostrava, Czech Republic; 4 Institute for Experimental Immunology, Affiliated to EUROIMMUN Medizinische Labordiagnostik AG, 23560 Lübeck, Germany; 5 Center for Health Research, Faculty of Medicine, University of Ostrava, 70300 Ostrava, Czech Republic

**Keywords:** COVID-19, SARS-CoV-2, cellular immunity, T-cells, humoral immunity, interferon-gamma release assay, IGRA

## Abstract

Cellular immunity against SARS-CoV-2 is an important component of the immune response to the virus. At present, two such tests based on interferon-gamma release (interferon-γ release assays, IGRAs) are available—Quan-T-Cell SARS-CoV-2 by EUROIMMUN and T-SPOT.COVID by Oxford Immunotec. In this paper, we compared the results of these two tests in 90 subjects employed at the Public Health Institute Ostrava who had previously undergone COVID-19 infection or were vaccinated against that disease. To the best of our knowledge, this is the first head-to-head comparison of these two tests evaluating T-cell-mediated immunity against SARS-CoV-2. In addition, we also evaluated humoral immunity in the same individuals using the in-house virus neutralization test and IgG ELISA assay. The evaluation yielded similar results for both IGRAs, with Quan-T-Cell appearing to be insignificantly (*p* = 0.08) more sensitive (all 90 individuals were at least borderline positive) than T-SPOT.COVID (negative results found in five patients). The overall qualitative (presence/absence of immune response) agreement of both tests with virus neutralization test and anti-S IgG was also excellent (close or equal to 100% in all subgroups, with the exception of unvaccinated Omicron convalescents, a large proportion of whom, i.e., four out of six subjects, were IgG negative while at least borderline positive for T-cell-mediated immunity measured by Quan-T). This implies that the evaluation of T-cell-mediated immunity is a more sensitive indicator of immune response than the evaluation of IgG seropositivity. This is true at least for unvaccinated patients with a history of being infected only by the Omicron variant, but also likely for other groups of patients.

## 1. Introduction

The COVID-19 pandemic sprouted unprecedented research efforts in the fields of virology and immunology. The virus causing this disease, SARS-CoV-2, has four principal structural proteins: the surface spike (S) protein, the envelope (E) protein, transmembrane (M) glycoprotein, and the internal phosphorylated nucleocapsid (N) protein [1]. The crucial virulence factor, i.e., the surface “spike” protein, consists of two subunits (S1 and S2). The S1 subunit serves to bind the virion to cellular receptors; its structure affects the cell and host tropism of the virus, while the role of the S2 glycoprotein is to aid the fusion of the virus with the cell membrane. The location of the spike protein on the viral surface makes it an important target for the immune system [2,3].

A viral infection provokes a complex immune response, including the development of immune memory. Adaptive immunity plays a crucial role in the resolution of most infections. The principal components of this system are B lymphocytes as effective tools of humoral (antibody-mediated) immunity and T lymphocytes constituting the main component of cellular immunity [4]. T-cells artificially transduced with chimeric antigen receptors have been also used in the treatment of COVID-19 [5].

Antibodies are produced in reaction to a viral infection or vaccination. In addition to a robust antibody response mediated through short-lived plasma cells during an active infection, long-lived plasma cells constitutively produce low levels of virus-specific antibodies providing long-term protection [6]. Still, antibody-mediated immunity is just a part of the response to a SARS-CoV-2 infection. Cellular immunity represented, in particular, by T-cells, is another crucial constituent of adaptive immunity and long-term immune memory [7,8], although it is often rather overlooked when discussing immunity against SARS-CoV-2. T-cell-mediated immunity precedes the antibody-mediated response. During a primary infection, specialized activated T-cells can be observed as soon as a few days after infection and also plays an important role during reinfection [9,10,11]. Thus, the evaluation of T-cell-mediated immunity against SARS-CoV-2 is of utmost importance as it might also serve as a possible predictor of protection from a severe course of future disease [12,13]. The detection of memory T-cells primed against SARS-CoV-2 by flow cytometry has been proposed as a highly specific method enabling the accurate identification of cellular phenotype [14], but the high technical requirements and costs are a downside of that method.

Therefore, the detection of effector T-cells primed to SARS-CoV-2 through interferon-γ release assays (IGRAs) has become probably the most widespread option for the evaluation of T-cell immunity, due to the commercial availability and simplicity of the testing procedure. Interferon-γ (IFN-γ) plays an important role in the mechanisms of both innate and adaptive immunity against viral infections, induces the synthesis of some enzymes leading to the suppression of viral replication, and regulates other significant aspects of the immune response [15,16]. IGRAs detect the production of IFN-γ by peripheral effector T-cells stimulated by antigens they have been previously exposed to.

Two such commercial platforms are currently available, both utilizing the principle of stimulation of effector cells from a blood sample with a specific antigen. The two methods, however, differ in the endpoint: one employs the spectrophotometric ELISA (enzyme-linked immunosorbent assay) detection of produced IFN-γ in plasma [17,18,19]; while the other method, ELISpot (enzyme-linked immunospot), allows the detection of IFN-γ production at the level of individual cells [20,21,22,23].

To the best of our knowledge, however, these two tests have never been compared in a head-to-head setting so far. For this reason, we undertook the presented study, which aimed to: (i) compare the performance of the two tests evaluating T-cell-mediated immunity against SARS-CoV-2; and (ii) analyze associations between the T-cell-mediated and humoral immunity.

## 2. Methods

### 2.1. Subjects and Sample Collection

Between August and October 2022, blood samples were collected from 92 Czech healthcare workers (Public Health Institute Ostrava, Ostrava, Czech Republic) who volunteered to participate in the study and signed informed consent. The study was approved by the Ethics Committee of the Public Health Institute Ostrava, protocol code number P01/2021.

Inclusion criteria were: age 18–75, history of laboratory-confirmed SARS-CoV-2 infection (PCR or antigen test dating back from 3 weeks to 2 years), and/or COVID-19 vaccination. Three blood collection tubes were taken from each participant: one tube (5 mL) for the determination of IgG and IgM antibody levels using ELISA and virus neutralization test (VNT, also known as plaque reduction test), and two tubes (2.5 mL and 5 mL of heparinized blood, respectively), for the determination of T-cell-mediated immunity. In two individuals, the result of one of the tests was invalid; hence, these individuals were removed from the study, bringing the total number of participants to 90.

Participants were classified into five subgroups according to the SARS-CoV-2 variant (based on the infection dates and the variant dominant at the time) and vaccination status: (i) unvaccinated Omicron convalescents; (ii) unvaccinated Wuhan/Alpha/Delta convalescents; (iii) unvaccinated individuals who suffered reinfection; (iv) vaccinated who tested SARS-CoV-2 positive in the past; and (v) vaccinated who have never tested positive.

### 2.2. T-Cell-Mediated Immunity

T-cell-mediated immunity was determined using two different IGRAs. The first evaluated IGRA, the **Quan-T-Cell SARS-CoV-2** by EUROIMMUN (Lübeck, Germany; hereinafter referred to as Quan-T), was performed in accordance with the manufacturer’s instructions as follows: 0.5 mL of heparinized whole blood was pipetted into three special antigen-containing incubation tubes (Quan-T-Cell SARS-CoV-2, catalog No. ET 2606-3003; EUROIMMUN) and incubated for 20–24 h at 37 °C to stimulate the T-cells. Subsequently, the blood was centrifuged (12,000× *g*), and the levels of produced IFN-γ were measured using the associated ELISA kit (Quan-T-Cell ELISA, catalog No. EQ 6841-9601; EUROIMMUN). Positive and negative controls were performed for each sample. The result was considered positive at values of >200 mIU/mL, borderline positive (100–200 mIU/mL), or negative (<100 mIU/mL).

**Figure 1:**
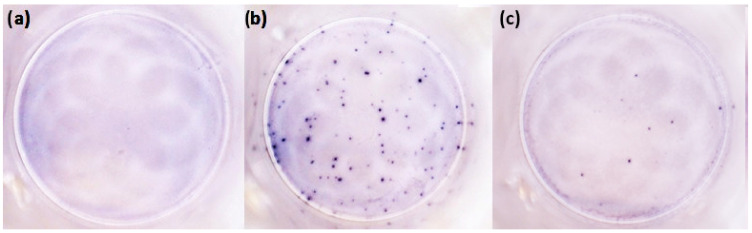
The evaluation of the T-Spot test: (**a**) negative; (**b**) positive; (**c**) borderline positive.

The other test, **T-SPOT.COVID** produced by Oxford Immunotec (Oxford, UK; hereinafter referred to as T-Spot; catalog No. COV.435/300) is a standardized ELISPOT-based technique intended for the qualitative detection of T-cell-mediated immune response to SARS-CoV-2 in heparinized human whole blood. Peripheral blood mononuclear cells (PBMCs) were separated from the whole blood by gradient centrifugation, washed, counted, and pipetted into 96-well microtiter plates (250,000 ± 50,000 PBMCs per well) with phytohemagglutinin and medium as positive and negative controls, respectively, and with two separate panels of SARS-CoV-2 antigens derived from the S and N proteins. The plate was incubated in a humidified incubator at 37 °C with 5% CO_2_ for 16–20 h. The secreted cytokine was captured by specific antibodies immobilized on the well bottom. After removing the plate from the incubator, the wells were washed and a second antibody, conjugated to alkaline phosphatase and directed to a different epitope on the cytokine molecule, was added to bind to the cytokine captured on the membrane surface. Any unbound conjugate was removed by washing and a soluble substrate was added to each well. The insoluble precipitate at the site of the reaction formed large, round, and dark spots at the site of the reaction. The result was then evaluated as follows: ≤4 spots indicated a negative result; 5–7 borderline positive; and ≥8 a positive result (see Figure 1). For each individual, three results were considered, namely the result of each panel N or S, respectively, and the overall T-Spot result (if the sample was positive in any of the panels, the overall T-Spot result was considered positive). The fact that this test separately evaluates response to the N and S proteins is an expected advantage of the T-Spot test, as mRNA or vector vaccines stimulate the response only to the S protein, the reaction to the N protein can be considered proof of past infection even in vaccinated individuals.

### 2.3. Humoral Immunity

IgG antibodies against the S protein (anti-S1 IgG) were semiquantitatively determined by ELISA using the kit Anti-SARS-CoV-2 QuantiVac ELISA IgG by EUROIMMUN (catalog No. EI 2606 10G), in accordance with the manufacturer’s instructions. The values were expressed as the positivity index (PI, also known as a signal-to-cut-off ratio, calculated as the ratio between the sample response and the response of the internal cut-off control; PI values < 0.9 indicate a negative result; 0.9–1.1 a borderline positive result; and >1.1 a positive result for the presence of antibodies in the sample) using calibrator 5.

An in-house virus neutralization test (VNT) indicating the antibody-mediated immune response was performed in sterile 96-well plates, as described in detail before [24,25]. In brief, the Delta strain of SARS-CoV-2 and CV-1 cells (African green monkey kidney fibroblasts) were used for testing. Serum samples were diluted in two-fold serial dilutions which, after mixing with the virus, resulted in final serum concentrations of 1/10, 1/20… up to 1/2560. These dilutions were mixed with the virus solution and incubated overnight at +4 °C, after which they were incubated with CV-1 cell suspension for a further 3–4 days at 37 °C and 5% CO_2_ atmosphere. Finally, the survival of the CV-1 cells was assessed using the neutral red dye. The virus neutralization titer was determined as the inverted value of the highest dilution of the sample neutralizing the cytopathic effect of the virus by more than 50%. Values below 10 were considered proof of the absence of any humoral neutralizing immunity against SARS-CoV-2 and values of ≥20 were considered positive.

### 2.4. Statistical Methods

The agreement between the T-Spot and Quan-T tests was evaluated using the test of symmetry and expressed as % of agreement with a 95% confidence interval. Correlations between the quantitative results of all tests were evaluated using the Spearman correlation coefficient (ρ). The level of significance was set to *p* = 0.05 for all tests. All computations were performed in Stata version 17. For the purposes of the correlation analyses, all VNT titers> 2560 were assigned the value of 2560, Quan-T values > 2500 were assigned the value of 2500, and T-Spot values (both S and N) > 20 were assigned the value of 20.

## 3. Results

### 3.1. Study Group

The final study group included 90 individuals from the Public Health Institute in Ostrava, Czech Republic. The mean age of participants was 48.9 ± 9.7 years (min. 25, max. 71 years). In total, 88% (N = 79) of participants were women, and 80% (N = 72) were vaccinated. The vast majority of vaccinated participants (65) were vaccinated with Comirnaty, three with the Janssen, two with the Vaxzevria, and one with the Spikevax vaccine, respectively. In total, 76% (N = 68) of participants had a personal history of a positive PCR test for COVID-19, of which 15 were reinfected. The median time from the last immunization impulse (SARS-CoV-2 infection or vaccination) was 274 days, with an interquartile range (IQR) of 209–307 days.

### 3.2. Comparison of the Quan-T and T-Spot Tests

**Figure 2:**
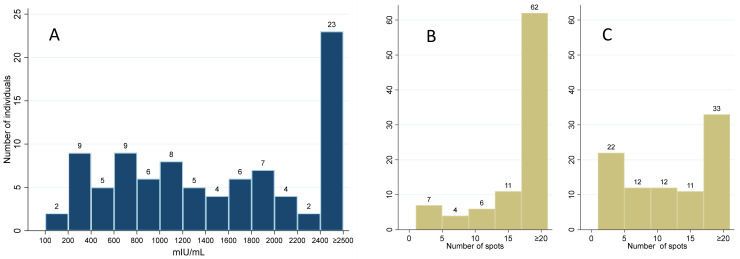
The distribution of the results of individual IGRA tests evaluating T-cell-mediated immunity: (**A**) Quan-T IGRA (<100 mIU/mL negative, 100–200 mIU/mL borderline positive, >200 mIU/mL positive) and T-SPOT.COVID with (**B**) spike, and (**C**) nucleocapsid antigen (≤4 negative, 5–7 borderline positive, ≥8 positive).

Out of the 90 samples, the Quan-T test identified 88 (97.8%) as positive and two as borderline positive, compared to five negatives, two borderline positives, and 83 (92.2%) positives detected by T-Spot. The T-Spot result can be further classified according to the individual antigens (spike and nucleocapsid antigens, respectively). The T-cell response to the spike antigen was absent in seven (7.8%) samples, borderline positive in four (4.4%), and positive in the remaining 79 (87.8%) samples, while the response to nucleocapsid antigen was negative in 22 (24.4%), borderline positive in nine (10.0%), and positive in 59 (65.6%) samples, respectively. The distribution of the results is presented in Figure 2.

Based on these results, the agreement between the T-Spot and Quan-T tests was 94.4% (95% CI: 87.5–98.2%), implying that there was no statistically significant difference between the methods (*p* = 0.082; see Table 1).

### 3.3. Correlations between Tests

Table 2 shows the correlations (Spearman correlation coefficients) between individual pairs of tests evaluating both humoral and cellular immunity against SARS-CoV-2 for individual categories of subjects. The correlation between VNT (neutralizing antibodies) and IgG levels was statistically significant in almost all groups of patients. Interestingly, it was almost perfect (ρ = 0.885) in unvaccinated convalescents, somewhat worse but still significant in vaccinated convalescents (ρ = 0.435), but insignificant in vaccinated previously uninfected individuals.

The overall correlation between the two T-cell-mediated immunity assays evaluating anti-S response was statistically significant. This applied also to most subcategories, except for unvaccinated convalescents (Table 2). The same can be said about the two T-Spot panels the anti-S and anti-N panels correlated for all groups, except for vaccinated convalescents.

Although both anti-S T-cell-mediated immunity tests showed a relatively weak overall correlation with IgG values, only Quan-T also correlated with the VNT test. Again, these correlations were more pronounced among convalescent patients than among only vaccinated patients but, interestingly, were not observed in unvaccinated convalescents.

### 3.4. Comparison of Humoral and Cellular Immunity Considering the Variant of the Past Infection

In addition to the above, we have also performed an additional analysis of subgroups considering also the variants of the disease. For this purpose, the study group was classified into five subgroups, as presented in Table 3.

Overall, anti-S IgG were detected in most subjects; the only exception was the group of unvaccinated Omicron convalescents, which showed a surprisingly low antibody response (especially considering that their infections were relatively recent). Still, all these individuals were at least borderline positive for T-cell-mediated immunity.

Neutralizing antibodies measured by VNT were present in all subjects, except for one unvaccinated individual. All vaccinated individuals were positive both for the IgG antibodies and in the VNT.

Where cellular immunity is concerned, the Quan-T assay detected T-cell-mediated immunity in all subjects, thus appearing slightly (though insignificantly, as shown in Table 1) more sensitive than the T-Spot assay (100% vs. 94.4% of positive subjects, respectively), differing in the reinfected unvaccinated and vaccinated uninfected groups.

The overall response in the T-Spot assay for the nucleocapsid-triggered reaction was lower than that triggered by the spike protein. This is not surprising, since the vaccinated uninfected patients could not develop anti-nucleocapsid protein immunity (S-based vaccines). Still, another interesting result was observed in the group of vaccinated individuals without known infection, in which 12 out of 22 individuals showed sensitivity to nucleocapsid antigens, which implies that they contracted SARS-CoV-2 infection at some point in the past.

## 4. Discussion

Adaptive immunity to viral diseases, including SARS-CoV-2, is conveyed by both T- and B-cells and provides long-term immunity against the virus, particularly against a severe course of the (re)infection [26]. In the initial phases of the SARS-CoV-2 epidemics, laboratory diagnostics particularly focused on the development of assays for testing the humoral response to the virus. At present, however, tests for the evaluation of T-cell-mediated immunity are also available. The presented study is, to the best of our knowledge, the first study comparing the performance of these two commercially available tests for the evaluation of this branch of immunity. In addition, we have also evaluated the humoral response and looked for associations between both types of immunity.

### 4.1. T-Cell-Mediated Immunity and Comparison of the Quan-T and T-Spot Tests

As IFN-γ is one of the key cytokines produced by T-cells, it is not surprising that its production is widely used for the evaluation of T-cell-mediated immunity. In our study, we used two IGRAs differing in the endpoints detecting the produced IFN-γ. One of these tests uses full plasma with an ELISA endpoint (Quan-T-Cell SARS-CoV-2 by EUROIMMUN), the other utilizes separated lymphocytes and the ELISpot endpoint detecting the IFN-γ production on the level of individual cells (T-SPOT.COVID, Oxford Immunotec, Abingdon, UK).

Although the principle of both tests is identical, the differences in their design make them relatively difficult to compare. Moreover, there is no gold standard for the measurement of T-cell-mediated immunity that could tell us which of the tests is more accurate [27]. ELISpot tests capture the released IFN-γ directly in the vicinity of the secreting cell; this prevents its dilution in the supernatant, binding to the receptors of neighboring cells, or degradation. ELISpot tests are, therefore, often reported to be more sensitive than conventional ELISA tests as the reaction of specific T-cells is unaffected by the variability of the reaction environment [28]. This expectation was, however, not confirmed in our study; Quan-T detected the T-cell-mediated immunity in all subjects (which likely corresponds to the reality as all subjects were vaccinated and/or convalescents), while T-Spot failed to reveal any T-cell response in five samples. This suggests an insignificantly higher sensitivity of Quan-T over T-Spot (Table 1). A similar result was reported by Phillips et al. [29], who compared T-Spot with an in-house PITCH ELISpot; they also reported negative results of T-Spot in several individuals in whom their in-house test detected T-cell-mediated immunity.

ELISpot testing, unlike Quan-T, uses a well-defined number of cells. This test is, therefore, also highly suitable for the detection of the T-cell response to vaccination in immunocompromised patients [30]. In this respect, it may be superior to conventional ELISA tests, in which an insufficient number of T-cells in the blood samples from such patients may lead to false negative results. We, however, can neither confirm nor dispute this statement as our study contained no immunocompromised patients.

Another T-Spot advantage over Quan-T lies in the possibility to evaluate effector cells reacting to stimulation by the N protein, which is a highly immunogenic antigen common to multiple coronaviruses, but the vaccine mRNA does not contain the sequence encoding this protein. Analogically to the antibodies against the N protein [31], the presence of T-cells reacting to stimulation by this antigen, therefore, implies that the respective individual had already been infected by SARS-CoV-2 in the past, not just vaccinated.

There are, however, also advantages of Quan-T over T-Spot. In addition to the aforementioned good sensitivity of Quan-T, other advantages include the simplicity of the procedure, or the possibility to incubate the whole blood with the antigen and then freeze the separated plasma for later evaluation. This supports better procedure planning and sample management in the laboratory. On the other hand, it must be taken into account that Quan-T is a functional test from full blood, and during incubation, individual cell types may also produce other cytokines (IL-2, IL-4, IL-5, IL 6, IL-10, IL-12, TNFα). These may, together with certain plasma proteins, form a stimulating environment, and, in turn, occasionally affect the results. In T-Spot, this effect is reduced (albeit not fully mitigated).

In all, our results showed that both tests provide similar results. Each of these tests has its pros and cons but both are viable options for the successful determination of T-cell-mediated immunity against SARS-CoV-2, and both appear to more sensitively indicate past infection or vaccination than simple IgG. In our study, this was particularly seen in unvaccinated individuals who were previously infected with the Omicron variant; despite their low IgG response, all were at least borderline positive in both tests evaluating T-cell response. T-cell activity against SARS-CoV-2 was also detected in all patients who were vaccinated, but never infected with the disease. This is also not surprising; as Schiavoni et al. have reported, vaccination induced Anti-SARS-CoV-2 T-cell activity even in individuals who were on immunosuppressant treatment during vaccination [32].

### 4.2. Humoral Immunity

The long-lived IgG and the assumed formation of memory B-cells capable of the rapid production of high-affinity Anti-SARS-CoV-2 IgG antibodies play a critical role in preventing the interaction of the virus with host cells, thus contributing towards viral clearance and to the control of disease progression [33].

In our study, we detected IgG antibodies in almost all subjects. The only groups standing out from this general trend were unvaccinated Omicron convalescents whose IgG responsiveness was relatively low. At first, we considered this to be caused by the fact that the spike protein of the Omicron variant evolved to such a degree that anti-S-IgG induced solely by the Omicron variant showed lower affinity in ELISA kits based on a previous variant of the virus [34]. For this reason, we have procured an ELISA kit for IgG detection designed specifically for the Omicron variant, namely the Anti-SARS-CoV-2 Omicron ELISA (IgG) produced by EUROIMMUN (catalog No. EI 2606-9601-30 G), and we tested the six samples from unvaccinated Omicron convalescents according to the manufacturer’s instructions using this kit. The results were, however, almost identical to those obtained using the original test we employed in this study. The lower level of IgG in these patients might, therefore, be due rather to the fact that the Omicron variant (generally) causes less severe disease than previous variants, and is largely contained on the mucosal surfaces and by cellular immunity. This does not necessitate the formation of large amounts of specific antibodies. Such an absence of antibodies has been observed after other viral infections in the past [9,10,35].

It was also interesting to see the almost perfect correlation (ρ = 0.885) between VNT and IgG levels in unvaccinated convalescents, dropping to 0.435 in vaccinated convalescents, and a mere 0.334 in vaccinated uninfected individuals. This is, however, in line with immunological principles, indicating a generally more complex stimulation of the immune system after infection than after vaccination only [36,37].

### 4.3. Associations between Humoral and Cellular Immunity

The results show that the Quan-T test is better correlated with results describing humoral immunity (both IgG and VNT) than the T-Spot test. This may be partially associated with the aforementioned stimulative environment in the Quan-T test caused by the fact that whole blood, containing also other immune factors (so-called bystander activation) [38], is used in this test. We can speculate that Quan-T better corresponds to the in vivo situation and, therefore, it is logical that it would be better correlated with the VNT test, which also mimics the in vivo situation. On the other hand, we see this better correlation of Quan-T over T-Spot also in the case of IgG, which should not be affected much by the presence of additional factors in the reaction mixture and, therefore, this explanation is likely responsible only for a part of the observed effect. Again, we can see better correlations between cellular and humoral immunity in convalescents compared to (only) vaccinated patients. This is likely caused by the more complex stimulation of the immune system by infection, compared to vaccination.

On the other hand, it is important to keep in mind that quantitative evaluation (expressed by correlations) is not as important as the binary agreement on the presence or absence of the particular class of immunity. The presence of any IgG antibodies (regardless of their amount) necessarily demonstrates the presence of memory B-cells capable of a rapid reaction to reinfection, and the production of large amounts of IgG to fight the infection. Similarly, the detection of any T-cell-mediated immunity indicates the presence of long-lived T-cells capable of rapid multiplication in the case of an infection. From this perspective, we can say that this agreement among all evaluated tests is very good to excellent (of course, we cannot consider the T-Spot N panel in this respect as, in principle, it cannot be positive in vaccinated truly uninfected individuals). This good qualitative agreement, despite poor quantitative correlation, has also been reported by Seraceni et al., who did not find any difference in T-cell response between the groups with low and high antibody levels [19]. On the other hand, Barreiro et al., who compared the concentrations of anti-S-IgG and IGRA results, found better correlations between the two branches of immunity, than our study [18]. This can be caused by the fact that their study evaluated the response in a cloistered community at approximately one month after an outbreak of COVID-19, at the time when all components of immunity against SARS-CoV-2 peaked. In our study, however, some individuals were as much as two years after their initial encounter with this virus, and waning immunity can play a role in this reduction of quantitative association.

It should be also noted that 12 out of 22 vaccinated patients who were never positively tested for SARS-CoV-2 were positive for the anti-N T-cell response, implying that they were through a SARS-CoV-2 infection. At the same time, this implies that the prevalence of past infection with SARS-CoV-2 is even higher than expected, based on seroprevalence studies [39].

### 4.4. Study Limitations

The principal limitation of our study lies in the absence of unvaccinated uninfected individuals, which would be necessary to perform a better evaluation of false positive/negative rates of the two tests. However, at the time of the experiment, there were literally no employees who had not been exposed to the SARS-CoV-2 antigens through the disease or vaccination, so we were unable to recruit such a group. Additionally, the subgroups were too small for any firm statements with respect to the immunogenicity of individual variants of SARS-CoV-2. In addition, the periods between the immunization impulse and sampling differed quite a lot among variants. For these reasons, we have to emphasize that the discussion on subgroups, although in our opinion valuable as it lays grounds for further research, cannot lead to any strong conclusions prior to being verified on larger groups. Lastly, the IgG test we used should normally be evaluated using the BAU, not positivity index; however, as the used correlation coefficient is non-parametric and, therefore, works with the order of the values rather than with the individual values, the results would not be affected by using linear scale BAU units instead of a non-linear one.

## 5. Conclusions

The following conclusions of our study, evaluating two standardized IGRA tests for SARS-CoV-2-induced T-cell-mediated immunity, can be used for future studies: (i) Both tests (Quan-T-Cell and T-SPOT.COVID) yielded similar results, with Quan-T appearing to be insignificantly more sensitive. On the other hand, the advantage of T-Spot lies in its capability of distinguishing between vaccine-induced and virus-induced immunity. Hence, where distinguishing between these two mechanisms of immunity development, ELISpot could be superior, while in everyday practice, the EUROIMMUN IGRA appears to be a better choice (among other things, due to the easier laboratory management). (ii) The agreement with indicators of humoral immunity (anti-S IgG and virus-neutralizing antibodies) is also very good if only binary response (immunity present/absent) is considered, although quantitative agreement represented by correlation coefficients is poorer, especially for T-Spot. Nevertheless, (iii) both IGRAs appear to be more sensitive than testing for IgG, especially in unvaccinated Omicron convalescents, and can be recommended to detect a past infection in patients without detectable antibodies, in immunocompromised patients, those on immunosuppressant treatment, for the detection of immune response in individuals with a low humoral response, or in immunocompetent individuals in whom IgG levels decreased over time after the infection or vaccination.

## Figures and Tables

**Table 1:** Qualitative comparison between results obtained using T-Spot and Quan-T IFN-γ release assays.

N = 90 Individuals (100%)		T-Spot (Combined S and N)		
		Positive	Borderline Positive	Negative
Quan-T	positive	81 (90.0%)	2 (2.2%)	5 (5.6%)
	borderline pos.	2 (2.2%)	0	0
	negative	0	0	0
*p*-value (Test of symmetry)		0.082		
Overall agreement *		94.4% (95% CI: 87.5–98.2%)		

* borderline positives are considered positives for the purposes of the calculation of the agreement; CI—confidence interval.

**Table 2:** Correlations (Spearman correlation coefficients) between individual tests of humoral and cellular immunity. Statistically significant correlations are highlighted in bold.

		Subgroup				
	All	All Vaccinated	All Convalescents	Unvaccinated Convalescents	Vaccinated Convalescents	Vaccinated Uninfected
*(Sub)group size (N)*	*90*	*72*	*68*	*18*	*50*	*22*
T-Spot S vs. T-Spot N	**0.373**	**0.541**	**0.271**	0.228	**0.472**	**0.542**
T-Spot S vs. Quan-T	**0.376**	**0.343**	**0.346**	−0.082	**0.208**	**0.545**
T-Spot S vs. IgG	**0.316**	0.146	**0.445**	0.126	0.183	0.173
T-Spot N vs. IgG	−0.117	−0.140	−0.017	0.321	−0.144	0.086
Quan-T vs. IgG	**0.454**	**0.304**	**0.497**	0.005	**0.321**	0.305
T-Spot S vs. VNT	0.092	−0.161	0.216	−0.033	−0.190	−0.026
T-Spot N vs. VNT	−0.172	−0.164	−0.058	0.147	−0.108	−0.081
Quan-T vs. VNT	**0.304**	0.090	**0.385**	−0.194	0.182	−0.056
IgG vs. VNT	**0.668**	**0.421**	**0.725**	**0.885**	**0.435**	0.334

**Table 3:** Subgroup analysis according to the infection variant; results of tests are presented as % positive (negative/borderline positive/positive); the borderline positives are considered positive for the purposes of the calculation of the overall positivity.

Group	N	Days from Last Immunization Impulse—Median (IQR)	Humoral Immunity		T-Cell-Mediated Immunity			
			IgG ELISA	VNT	Quan-T	T-Spot (Total)	T-Spot (S)	T-Spot (N)
Omicron unvaccinated	6	209	**33.3%**	**100%**	**100%**	**100%**	**83.3%**	**83.3%**
		(138–221)	(4/2/0)	(0/2/4)	(0/1/5)	(0/0/6)	(1/1/4)	(1/1/4)
Wuhan/Alpha/Delta unvaccinated	6	315 (310–616)	**83.3%** (1/0/5)	**83.3%** (1/0/5)	**100%** (0/0/6)	**100%** (0/0/6)	**83.3%** (1/0/5)	**100%** (0/2/4)
Reinfected unvaccinated	6	263	**100%**	**100%**	**100%**	**66.6%**	**66.6%**	**50.0%**
		(201–316)	(0/0/6)	(0/0/6)	(0/0/6)	(2/0/4)	(2/0/4)	(3/0/3)
Vaccinated convalescents	50	238	**100%**	**100%**	**100%**	**100%**	**100%**	**84.0%**
		(204–307)	(0/0/50)	(0/0/50)	(0/1/49)	(0/1/49)	(0/1/49)	(8/4/38)
Vaccinated uninfected	22	286	**100%**	**100%**	**100%**	**86.4%**	**86.4%**	**54.5%**
		(284–304)	(0/0/22)	(0/0/22)	(0/0/22)	(3/1/18)	(3/2/17)	(10/2/10)
Total	90	284	**94.4%**	**98.9%**	**100%**	**94.4%**	**92.2%**	**75.6%**
		(209–307)	(5/2/83)	(1/2/87)	(0/2/88)	(5/2/83)	(7/4/79)	(22/9/59)

N–number of subjects; VNT–virus neutralization test; assignment of the SARS-CoV-2 variants is conjectural (derived from the date of PCR positivity); IQR–interquartile range.

## Data Availability

The raw data are available from the authors upon reasonable request.
